# Assessing pressure wave components for aortic stiffness monitoring through spectral regression learning

**DOI:** 10.1093/ehjopen/oeae040

**Published:** 2024-05-21

**Authors:** Arian Aghilinejad, Morteza Gharib

**Affiliations:** Division of Engineering and Applied Science, California Institute of Technology, 1200 E California Blvd, Pasadena, CA 91125, USA; Division of Engineering and Applied Science, California Institute of Technology, 1200 E California Blvd, Pasadena, CA 91125, USA

**Keywords:** Blood pressure, Haemodynamics, Aortic stiffening, Pulse wave analysis

## Abstract

**Aims:**

The ageing process notably induces structural changes in the arterial system, primarily manifesting as increased aortic stiffness, a precursor to cardiovascular events. While wave separation analysis is a robust tool for decomposing the components of blood pressure waveform, its relationship with cardiovascular events, such as aortic stiffening, is incompletely understood. Furthermore, its applicability has been limited due to the need for concurrent measurements of pressure and flow. Our aim in this study addresses this gap by introducing a spectral regression learning method for pressure-only wave separation analysis.

**Methods and results:**

Leveraging data from the Framingham Heart Study (2640 individuals, 55% women), we evaluate the accuracy of pressure-only estimates, their interchangeability with a reference method based on ultrasound-derived flow waves, and their association with carotid-femoral pulse wave velocity (PWV). Method-derived estimates are strongly correlated with the reference ones for forward wave amplitude ($R^{2}=0.91$), backward wave amplitude ($R^{2}=0.88$), and reflection index ($R^{2}=0.87$) and moderately correlated with a time delay between forward and backward waves ($R^{2}=0.38$). The proposed pressure-only method shows interchangeability with the reference method through covariate analysis. Adjusting for age, sex, body size, mean blood pressure, and heart rate, the results suggest that both pressure-only and pressure-flow evaluations of wave separation parameters yield similar model performances for predicting carotid-femoral PWV, with forward wave amplitude being the only significant factor (*P* < 0.001; 95% confidence interval, 0.056–0.097).

**Conclusion:**

We propose an interchangeable pressure-only wave separation analysis method and demonstrate its clinical applicability in capturing aortic stiffening. The proposed method provides a valuable non-invasive tool for assessing cardiovascular health.

## Graphical Abstract

**Graphical Abstract:**
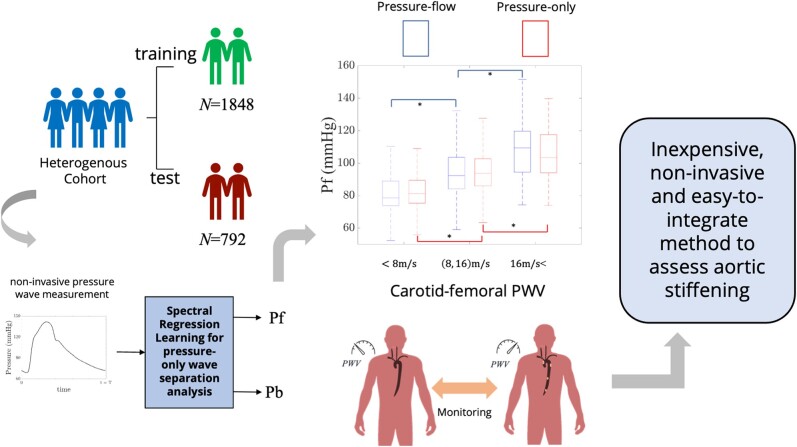

## Introduction

Aortic stiffness increases with age and is one of the earliest pathological changes within the arterial wall, affecting the wave dynamics in the vasculature.^1–8^ Nevertheless, there remains considerable ambiguity regarding the contributions of various components of blood pressure waveform and wave reflection to aortic stiffness. In classical pressure wave analysis, wave reflection is frequently assessed through the augmentation index (AIx).^9^ The clinical significance of AIx has been highlighted in recent work by Yofoglu *et al*.,^10^ demonstrating its correlations with left ventricle mass. However, a limitation of AIx lies in its dependency not solely on the magnitude but also on the timing of wave reflection.^9,11^ This timing is influenced by a range of physical and physiological factors, including subjects’ height and heart rate.^11,12^ To provide a more comprehensive evaluation of wave reflection, wave separation analysis has been employed.^13^ This method decomposes the pressure pulse into a forward pressure wave, travelling from the heart to the periphery, and a reflected pressure wave, travelling backward towards the heart.^14,15^ Zamani *et al*.^16,17^ highlighted the clinical significance of wave separation analysis by showing its ability to correlate with all-cause mortality in individuals initially free of clinically evident cardiovascular disease. In the Framingham Heart Study (FHS), Cooper *et al*.^18^ associated forward pressure wave amplitude with incident cardiovascular disease, whereas mean arterial pressure and global wave reflection did not show similar associations.

Despite the compelling evidence supporting the importance of wave separation analysis, its full integration into clinical practice is hindered by the requirement for simultaneous measurements of pressure and flow waveforms. Although approximate approaches based on triangular flow profiles or Windkessel-based methods enable wave separation analysis using only pressure measurements, implementing these protocols in clinical settings is challenging, and their applicability has faced difficulties in large heterogeneous populations.^12,19–22^ Notably, the study by Kips *et al*.^23^ in the Asklepios population suggested substantial differences in results between pressure-based approximative methods and those using both pressure and flow information.

In this study, utilizing data from the population-based FHS, our objectives are to: (i) evaluate the accuracy of pressure-only estimates for wave separation parameters through the application of the proposed spectral regression learning method, (ii) conduct a covariate analysis to investigate the interchangeability between the proposed pressure-only method and the reference method, and (iii) investigate the association between wave separation parameters (using the reference as well as the proposed pressure-only method) and aortic stiffness, as measured by carotid-femoral pulse wave velocity (PWV). The selection of carotid-femoral PWV as a validation metric is based on its crucial role in governing wave dynamics within the cardiovascular system, along with its well-established pathophysiological association with the early arrival of pressure wave reflections.^24–28^

## Methods

### Participants and data

In this investigation, we employed data from the FHS, a population-based epidemiological cohort analysis. Details are provided in the previous works.^15,18,28–30^ The sample was drawn from the eight-examination cycle of an offspring cohort. The characteristics of the participants, including a heterogeneous cohort of *n* = 2640 individuals (comprising 1201 males and 1439 females, aged between 40 and 91 years), are presented in *Table 1*. All participants provided written informed consent, and the study protocols received approval from the Boston University Medical Campus and Boston Medical Center Institutional Review Board. Initially, the clinical data set is split into the training and testing data for all regression learning analyses. The models are strictly trained on the training population, and the testing data set is used only once to evaluate the accuracy of the model. The characteristics of these two data sets are also presented in *Table 1*. All participants underwent a thorough and non-invasive evaluation of central haemodynamics, resulting in a comprehensive collection of tonometry recordings for carotid pressure waveforms. Aortic flow waveforms were acquired through two-dimensional echocardiography of the left ventricular outflow tract, followed by pulsed Doppler from an apical five-chamber view to acquire the aortic flow waveform. Additionally, tonometry data were digitized at a rate of 1000 Hz during the primary acquisition process, and the waveforms were signal-averaged using the electrocardiogram R-wave as a fiducial point. Calibrated carotid pressure served as a surrogate for central pressure. Carotid-femoral PWV was used as a metric for aortic stiffness, as previously described.^15,18,31^

**Table 1:** Baseline characteristics of total patient data (*n* = 2640), the training subpopulation (*n* = 1848), and the testing subpopulation (*n* = 792)

Variable	Total (n=2640)	Training (n=1848)	Test (n=792)
Age, years	66 ± 9	66 ± 9	66 ± 9
Women, *n* (%)	1439 (55)	1000 (54)	439 (55)
Height, cm	167 ± 10	167 ± 10	167 ± 9
Weight, kg	78 ± 17	79 ± 18	78 ± 17
Body mass index, kg/m^2^	27.9 ± 5.1	27.9 ± 5.1	27.9 ± 5.1
Heart rate, b.p.m.	62 ± 10	62 ± 10	62 ± 10
Brachial blood pressure, mmHg			
Systolic	141 ± 20	140 ± 20	141 ± 20
Diastolic	69 ± 9	69 ± 9	69 ± 9
Pulse	72 ± 19	71 ± 19	72 ± 18

All values are (mean ± standard deviation) except as noted.

### Wave separation analysis

Wave separation analysis, which facilitates the breakdown of arterial pressure waves into forward and backward wave components, has been previously outlined.^32^ This approach involves quantifying information about forward and backward waves through principles of fluid dynamics in compliant tubes.^12,33–36^ Flow and pressure data were utilized to compute forward and backward pressure waveforms using the linear wave separation technique, initially described by Westerhof *et al*.,^32^ and details are provided in the Supplementary material. The input impedance of the artery is calculated using Fourier analysis as the ratio of *P*(*t*) and *Q*(*t*) harmonics in the frequency domain.^15^ To evaluate the time delay (TD) between forward pressure wave amplitude (Pf) and backward pressure wave amplitude (Pb), the method introduced by Qasem and Avolio^22^ is used.

### Wave separation analysis via spectral regression learning

A pressure-only estimation of wave separation can be achieved through the hybrid spectral regression learning methodology introduced by Aghilinejad *et al*.,^37^ and further details are provided in the Supplementary material. In this approach, Fourier series (spectral) decomposition is employed for input feature selection. Initially, the carotid pressure waveforms from tonometry measurements are sampled at a rate of 1000 Hz, resulting in 1000 data points per single pressure measurement in a cycle size of 1 s. The high dimensionality of the input signal poses limitations on naive regression model constructs for practical applications. To address this, in our study, we utilized Fourier-based spectral analysis, specifically employing the Fast Fourier Transform (FFT), to transform data from a high-dimensional space to a low-dimensional one. This approach preserves meaningful properties of the original data, while reducing input dimensionality. The advantage of using FFT-based input reduction lies in the fact that high-frequency components do not provide significant additional physiological information for computing the wave separation parameters.^13^

**Figure 1:**
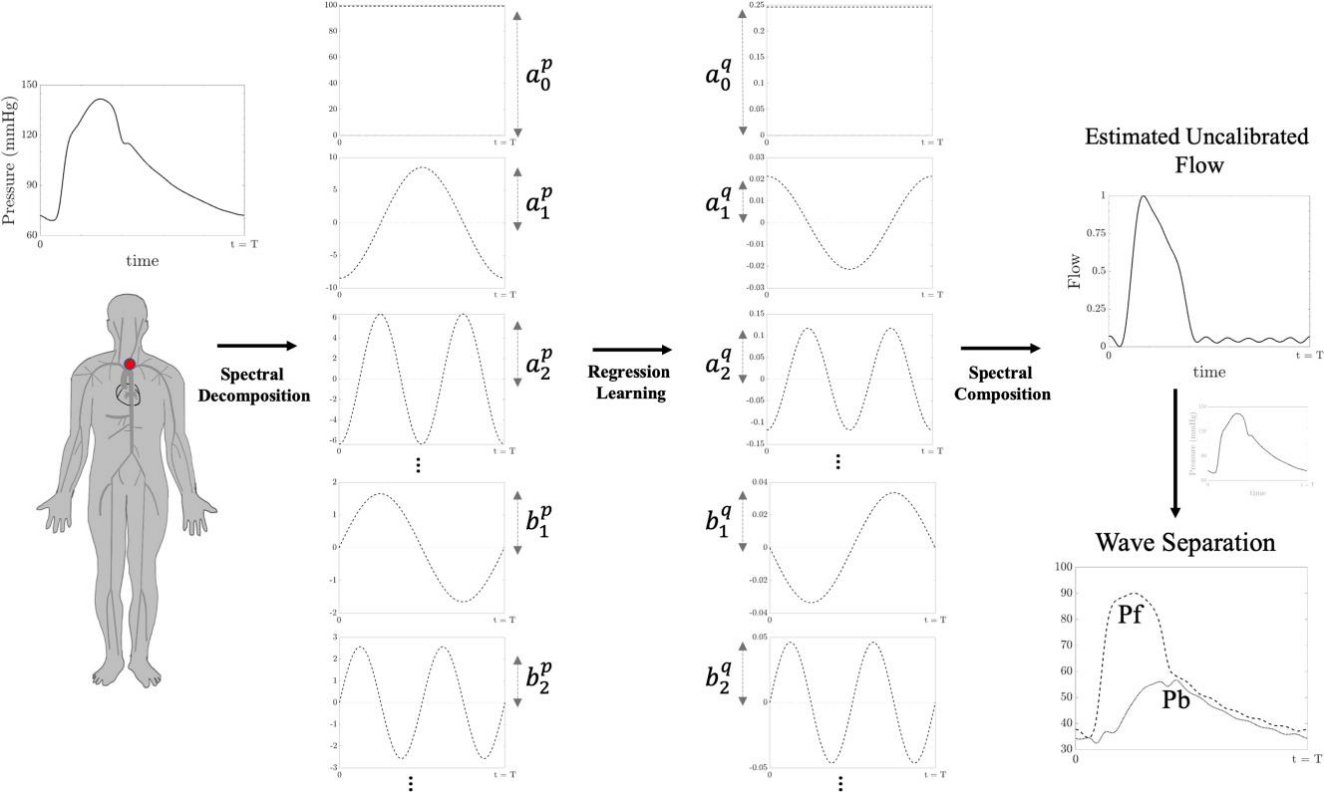
A description of the proposed spectral regression learning approach for a pressure-only wave separation analysis. The process starts with a non-invasive pressure waveform measurement, followed by a Fourier-based decomposition of the waveform. The wave components of the flow profile are then estimated by regression learning and then composed to reconstruct the uncalibrated flow profile. The estimated flow waveform, along with the measured pressure waveform, will be used to conduct a wave separation analysis.

The regression model is subsequently trained on features derived from the Fourier decomposition of the pressure waveform. These models are trained with the Fourier modes of the central pressure waveforms as input and the Fourier modes of the central flow waveforms as output. Following training, the testing data set is utilized once to assess the accuracy of the model. The carotid waveform in the testing data set undergoes spectral mode decomposition and is then input to the regression model. The output of the regression models, representing the estimated modes of the corresponding central flow waveform, is inverse-Fourier-transformed to the time domain using the computed modes and the length of the signal. As demonstrated in previous studies, there is no need to calibrate the flow profile.^21^ The estimated flow waveform from the regression model is then used to conduct the wave separation analysis. The steps for the model implementation are presented in *Figure 1*. In addition to the proposed wave separation analysis of this study, we also conducted pressure-only wave separation based on the generic triangular flow profile approximation^20,21^ for comparison in this study.

### Statistical analysis

*Table 1* illustrates the baseline characteristics of the study sample, with continuous variables from the sample data summarized as mean ± standard deviation (SD). Key parameters, including Pf, Pb, reflection index (RI), and TD between forward and backward pressure waves, were selected to assess the efficacy of the proposed pressure-only wave separation analysis. The reference values for wave separation in all cases were determined using central flow measurements in conjunction with carotid pressure measurements, serving as a surrogate for central pressure. To evaluate the accuracy of the proposed method, we compared the estimated values with those obtained through an exact wave separation analysis using Pearson correlation coefficients (*r*), the coefficient of determination, and root mean square errors. The agreement and bias between the exact wave separation variables and the estimated ones were further examined using a Bland–Altman analysis, presenting mean differences along with limits of agreement (mean bias ± 1.96 SD of the differences). Multivariate regression was used to explore the influence of clinical covariates on each wave separation variable as well as aortic stiffness (quantified by carotid-femoral PWV). The proportion of variability in the dependent variable explained by the model was presented as $R^{2}$. Additionally, regression coefficients (beta coefficients) were reported along with their 95% confidence intervals. Continuous variables were compared between the groups using the Kruskal–Wallis rank-sum test. Statistical significance was defined as *P* < 0.001. All mathematical and statistical analyses of the clinical data were performed using custom-written codes implemented in Python (Python Software Foundation, Python Language Reference, version 3.11).

## Results

### Accuracy of wave separation parameters

**Figure 2:**
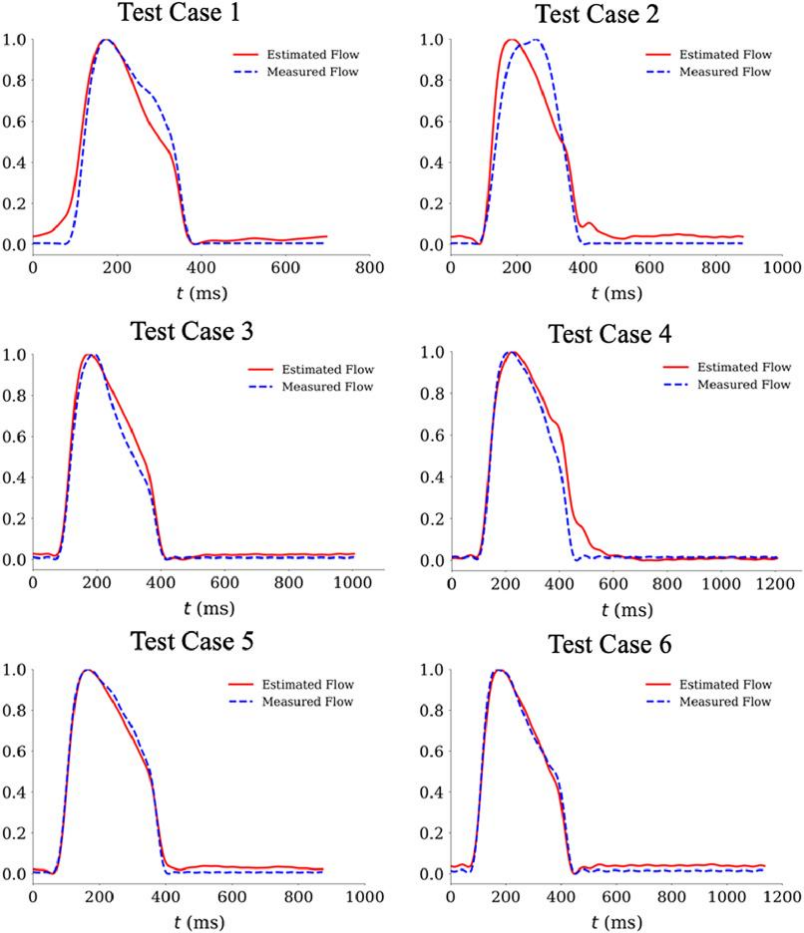
Typical sample cases of the estimated and measured central flow waveform profile. The blue waveform (dashed line) demonstrates the measured flow profile using ultrasound and the red one (solid line) demonstrates the estimated one using the spectral regression learning method.

*Table 2* presents correlations and errors between pressure-only estimates of wave separation parameters and reference values, as well as measures of RI (an indicator of the relative significance of backward pressure wave amplitude) and TD (an indicator of the time lag between the forward and the backward wave components of the pressure waveform). These results are demonstrated within the testing subset of the initial population. From the initial testing population of 792 individuals, 39 patients were excluded due to the failure in PWV measurement, resulting in 753 subjects in *Table 2*. The last column presents estimates derived based on flow estimation from the spectral regression learning proposed in this study. The accuracy of the uncalibrated estimated flow profiles, used to conduct pressure-only wave separation through spectral regression learning, is presented in Supplementary material online, *Table S1*. *Figure 2* illustrates six sample cases from the blind testing set, where the uncalibrated flow profile is estimated using spectral regression learning and overlaid on the measured flow profile. Supplementary material online, *Figure S1* demonstrates scatter and Bland–Altman plots indicating the agreement between the measured and the estimated uncalibrated averages of the mean flow profile, revealing the method’s ability to capture the shape of the flow profile. In *Table 1*, the estimated pressure-only wave separation parameters using conventional triangular flow estimation are also provided for comparison. In Supplementary material online, *Table S2*, average values for all wave separation parameters in different age groups based on pressure and flow measurements are tabulated for reference.

**Table 2:** Mean values, errors, and correlations between pressure-only estimates of wave separation parameters and reference values (*n* = 753)

Variables	Reference (pressure and flow)	Triangular flow estimation	Pressure-only estimation of this study
Pb (mmHg)	56 (8.2)	67 (10.4)	56 (8.3)
NRMSE (Pb^exact^ − Pb^est^) (%)	—	21.6	5.4
r (Pb^exact^ vs. Pb^est^)	—	0.94	0.94
Pf (mmHg)	92 (16.6)	91 (18.8)	93 (14.3)
NRMSE (Pf^exact^ − Pf^est^) (%)	—	7.5	4.8
r (Pf^exact^ vs. Pf^est^)	—	0.91	0.96
RI	0.38 (0.03)	0.43 (0.04)	0.38 (0.02)
NRMSE (RI^exact^ − RI^est^) (%)	—	25.5	5.4
r (RI^exact^ vs. RI^est^)	—	0.74	0.93
TD (ms)	64 (19)	63 (28)	64 (18)
NRMSE( TD^exact^ − TD^est^) (%)	—	30.5	15.9
r (TD^exact^ vs. TD^est^)	—	NC	0.62

NRMSE indicates the normalized root mean square errors by the range of the variable. The values given in the parentheses denote standard deviation. NC indicates not correlated (*r* < 0.2).

**Figure 3:**
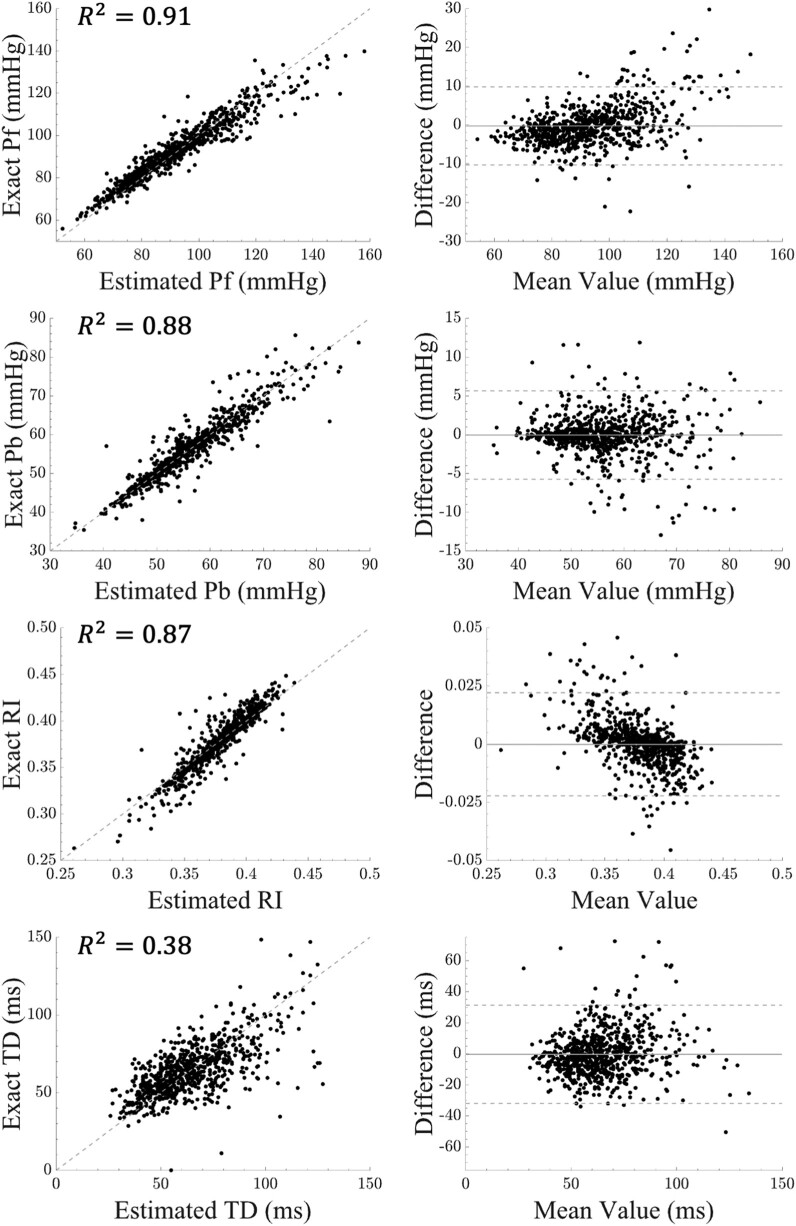
Scatter and Bland–Altman plots for wave separation parameters. The parameters are forward pressure wave amplitude (Pf), backward pressure wave amplitude (Pb), reflection index (RI), and time delay (TD) between forward and backward pressure wave amplitudes. The plots are demonstrated for the test data (n = 753).

*Figure 3* illustrates scatter and Bland–Altman plots indicating agreement in forward and backward pressure wave amplitudes between measured (using pressure and flow) and estimated (using pressure-only spectral regression learning) values. The coefficient of determination is also presented for all the wave separation parameters in these plots. The distributions of the residuals between reference and estimated variables with respect to age are also presented in Supplementary material online, *Figure S2*.

### Analysis of covariates for wave separation parameters

*Table 3* presents a regression analysis of covariates for measured (reference) and estimated forward and backward pressure wave amplitudes. Values for backward pressure wave amplitude (Pb) are independently related to age, height, heart rate, and mean blood pressure (*R*^2^ = 0.904). Weight and sex do not contribute significantly (*P* > 0.001). The estimated pressure-only models for backward pressure wave amplitude using spectral regression learning show similar results to the measured values (*R*^2^ = 0.873), except in terms of the significance level for height. An analysis of covariates for forward pressure wave amplitude suggests that values for Pf are independently related to age and mean blood pressure (*R*^2^ = 0.551). Heart rate, height, weight, and sex do not contribute significantly to forward pressure wave amplitude. The estimated pressure-only model for forward pressure wave amplitude suggests similar results to the same significant parameters (i.e. age and mean blood pressure; *R*^2^ = 0.627). Both types of models (based on measured and estimated wave separation parameters) show that the proportion of variance in wave separation variables based on common physiological parameters is better explained for backward pressure wave amplitudes (Pb) than forward pressure wave amplitudes (Pf).

**Table 3:** Regression analysis of covariates for measured (reference) and estimated forward (Pf) and backward (Pb) pressure wave amplitudes (*n* = 753)

Variables	β	SE (*β*)	CI (*β*)	*P*-value
Pb (mmHg) derived from both pressure and flow measurements, adjusted *R*^2^ = 0.904				
Age, years	0.039	0.011	(0.017, 0.062)	<0.001
Height, cm	−0.143	0.041	(−0.224, −0.063)	<0.001
Heart rate, b.p.m.	−13.03	0.571	(−14.15, −11.91)	<0.001
Mean blood pressure, mmHg	0.644	0.008	(0.629, 0.660)	<0.001
Pb (mmHg) derived from the proposed pressure-only measurement, adjusted *R*^2^ = 0.873				
Age, years	0.095	0.013	(0.069, 0.121)	<0.001
Height, cm	−0.089	0.047	(−0.183, 0.004)	<0.01^a^
Heart rate, b.p.m.	−8.86	0.664	(−10.166, −7.558)	<0.001
Mean blood pressure, mmHg	0.638	0.009	(0.621, 0.657)	<0.001
Pf (mmHg) derived from both pressure and flow measurements, adjusted *R*^2^ = 0.551				
Age, years	0.506	0.049	(0.406, 0.602)	<0.001
Height, cm	−0.171	0.178	(−0.526, 0.175)	<0.5^a^
Heart rate, b.p.m.	−5.175	2.493	(−10.10, −0.308)	<0.1^a^
Mean blood pressure, mmHg	0.901	0.035	(0.833, 0.971)	<0.001
Pf (mmHg) derived from the proposed pressure-only measurement, adjusted *R*^2^ = 0.627				
Age, years	0.388	0.039	(0.312, 0.465)	<0.001
Height, cm	−0.173	0.140	(−0.448, 0.102)	<0.5^a^
Heart rate, b.p.m.	−4.735	1.958	(−8.579, −0.892)	<0.1^a^
Mean blood pressure, mmHg	0.858	0.027	(0.805, 0.912)	<0.001

CI, confidence interval; SE, standard error.

^a^A non-significant parameter.

### Wave separation associations with aortic stiffening

**Figure 4:**
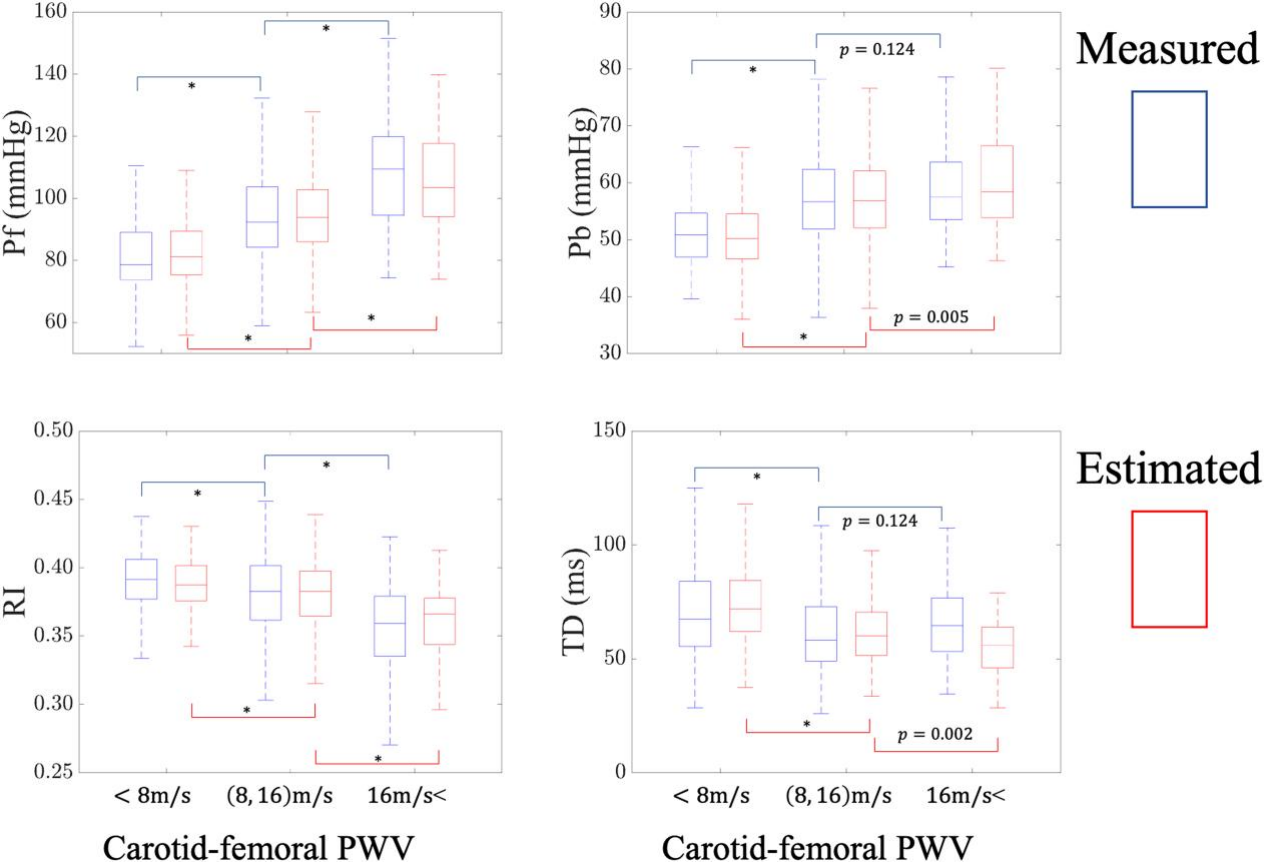
Boxplots for wave separation parameter distributions for different levels of aortic stiffness. Unadjusted comparisons of forward pressure wave amplitude (Pf), backward pressure wave amplitude (Pb), reflection index (RI), and time delay (TD) between participants with carotid-femoral pulse wave velocity <8, 8–16, and >16 m/s. Wave separation parameters are determined using the reference (pressure and flow) and spectral regression learning (pressure-only) methods.

*Figure 4* presents unadjusted analysis comparing measures of aortic stiffness in various carotid-femoral PWV groups. In this analysis, subjects with elevated aortic stiffness demonstrate a higher forward pressure amplitude compared with those with lower levels of aortic stiffness (*P* < 0.001), whereas the increase in backward pressure wave amplitude is less significant, especially between the group with a carotid-femoral PWV of 8–16 m/s and the one with carotid-femoral PWV >16 m/s. The between-group comparison is similar using either estimated or measured wave separation parameters. The RI is lower for participants with higher levels of aortic stiffness (*P* < 0.001), while the change in time delay is not significant between the group with a carotid-femoral PWV of 8–16 m/s and the one with carotid-femoral PWV >16 m/s.

*Table 4* presents the statistical contribution of forward, backward, and TD between pressure waves to carotid-femoral PWV based on measured (using pressure and flow measurements) and estimated (using pressure-only spectral regression learning) wave separation values. A base model not including the wave separation variables is presented as Supplementary material online, *Table S3* (*R*^2^ = 0.383). When forward pressure wave amplitude enters the model, the *R*^2^ increases to 0.428 for the model with an exact forward wave amplitude (based on pressure and flow measurement) and *R*^2^ increases to 0.413 for the model with estimated forward pressure wave amplitude (based on pressure-only measurement). When the Pb and time delay enter the model (as demonstrated in *Table 4*), increments to the model *R*^2^ are not significant.

**Table 4:** Haemodynamic correlates of the carotid-femoral pulse wave velocity using reference and pressure-only estimates of wave separation parameters (*n* = 753)

Variables	*β*	SE (*β*)	CI (*β*)	*P*-value
Model for carotid-femoral PWV based on the measured WSA, adjusted *R*^2^ = 0.430				
Sex	1.113	0.315	(0.495, 1.732)	<0.001
Age, years	0.177	0.014	(0.149, 0.204)	<0.001
Mean blood pressure, mmHg	0.059	0.029	(0.002, 0.116)	<0.05^a^
Heart rate, b.p.m.	2.715	0.852	(1.041, 4.389)	<0.01^a^
Pf, mmHg	0.077	0.010	(0.056, 0.097)	<0.001
Pb, mmHg	−0.094	0.046	(−0.184, −0.004)	<0.05^a^
TD, ms	−4.502	7.505	(−19.235, 10.232)	<0.6^a^
Model for carotid-femoral PWV based on the proposed pressure-only WSA, adjusted *R*^2^ = 0.414				
Sex	1.047	0.346	(0.422, 1.672)	<0.001
Age, years	0.178	0.014	(0.151, 0.206)	<0.001
Mean blood pressure, mmHg	0.019	0.025	(−0.030, 0.069)	<0.5^a^
Heart rate, b.p.m.	3.463	0.751	(1.989, 4.937)	<0.001
Pf, mmHg	0.081	0.014	(0.054, 0.108)	<0.001
Pb, mmHg	−0.042	0.044	(−0.129, 0.045)	<0.5^a^
TD, ms	−13.061	7.954	(−28.676, 2.554)	<0.2^a^

CI, confidence interval; PWV, pulse wave velocity; SE, standard error; TD, time delay between the forward and the backward waves; WSA, wave separation analysis, respectively.

^a^A non-significant parameter.

## Discussion

In this study, we comprehensively investigate the accuracy of a novel wave separation analysis using spectral regression learning and single pressure waveform measurements and investigate the associations with aortic stiffness. A primary finding of our study is that the wave separation parameters, estimated through the proposed spectral regression learning approach, outperform those based on the triangular flow approximation used conventionally in previous studies.^21^ This is due to the ability of our approach in capturing flow waveform morphology, as demonstrated in *Figure 2* and Supplementary material online, *Table S1*. *Table 2* and *Figure 3* indicate a significant correlation between forward pressure wave amplitudes and the reference forward pressure wave amplitude (*R*^2^ of 0.91) with negligible systemic bias. The normalized error associated with the spectral regression learning method for forward pressure wave amplitude is 5.4%, which is considerably better than the 21.6% error associated with the triangular flow approximation method. While the spectral regression learning method outperforms the triangular flow approximation method in terms of Pb, the difference in the error is less significant (4.8 vs. 7.5%). Following Qasem and Avolio,^22^ we also assess the TD between forward and backward pressure waves using a cross-correlation technique. Our results also suggest that, unlike the triangular flow approximation method, the proposed spectral regression learning method in this study provides accurate estimates of the TD between forward and backward pressure waves, with a correlation as high as 0.62 and a moderate *R*^2^ of 0.38. These results align with findings from other studies, such as the one by Kips *et al*.,^23^ which demonstrated that TD could not be accurately captured using the triangular flow approximation method in a large population due to an overestimation in wave components. Our approach utilizes Fourier-based wave decomposition to analyse pressure signals, enabling an accurate estimation of flow wave morphology and a subsequent separation of pressure waves. By leveraging harmonic content, we effectively decode the periodic and oscillatory waves present in the cardiovascular system. While previous methods, such as those employing multi-Gaussian decomposition,^38^ have shown efficacy in smaller data sets or virtual ones, our approach stands as one of the few to be successfully applied in a large, diverse cohort, building upon the valuable contributions of existing research.

The results further suggest that the regression analysis of covariates and determinants reveals no significant difference between the reference wave separation parameters and those derived from the pressure-only estimates using the spectral regression learning method (*Table 3*). These results indicate that the variation in backward pressure wave amplitude is well explained by age, heart rate, height, and mean blood pressure for both the reference and the estimated methods, with *R*^2^ values of 0.904 and 0.873, respectively. Although the *R*^2^ values based on the same covariates are smaller for forward pressure wave amplitude, there is no significant difference between the measured reference and the estimated values for forward pressure wave amplitude (*R*^2^ of 0.551 and 0.627, respectively). Previous studies have demonstrated that forward pressure wave amplitude serves as a measure of proximal aortic geometry and stiffness, while mean arterial pressure and backward (reflective) wave amplitudes are more correlated with the resistance of vessel structure and function.^18^ Consequently, the inclusion of mean arterial pressure as a determinant in the multivariate regression significantly improves the Pb model more than the forward pressure wave amplitude, thereby explaining the higher *R*^2^. The resemblance in the covariate regression models for both Pf and Pb between the reference and the estimated values implies a comparable physiological interpretation of the parameters obtained through the spectral regression learning method and the measured ones. It is worth noting that Hametner *et al*.^20^ demonstrated that drawing a similar conclusion is not possible when employing the triangular flow approximation or average flow method for pressure-only wave separation.

In our current investigation into the associations of wave separation parameters with aortic stiffness, participants were categorized into groups with carotid-femoral PWV <8, 8–16, and >16 m/s (*Figure 4*). Both the reference and the estimated measures of the wave separation parameters yielded consistent trends; forward pressure wave amplitudes differed significantly among the different groups (*P* < 0.001), while the backward pressure wave component and the time delay did not exhibit the same variations. Changes in the RI were also significant among different groups, yet this change could be attributed solely to the forward component. Additionally, we employed a multivariate model considering both Pf and Pb, as well as the TD between these two waves (*Table 4*). The results indicated that forward pressure wave amplitude remained significant, whereas Pb and TD did not. These findings are consistent with prior studies suggesting that forward wave amplitude is associated with the pulsatile load in the cardiovascular system, while Pb is more associated with the steady load component.^13,18,39^ Our findings further suggest that incorporating forward pressure wave amplitude into the base model enhances the model’s *R*^2^ using reference measures. By utilizing the estimate of forward pressure wave amplitude from the spectral regression learning method, the model’s *R*^2^ improves analogously. These results suggest that forward pressure wave amplitude plays a role in elevating carotid-femoral PWV (an indicator of vascular stiffening), and these adverse effects can be effectively captured using the proposed spectral regression learning method for wave separation based solely on pressure measurements. Ultimately, this platform provides valuable information for assessing cardiovascular health that can be incorporated in a wide range of non-invasive, inexpensive, and easy-to-use devices.^40–45^

### Study limitations and future work

The major limitation in this study is that we do not have invasively measured aortic pressure waveforms for determining the exact central pressure waveform. Future studies employing invasive clinical measurements can further expand the applicability of the proposed spectral regression learning for the wave separation analysis. However, our choice of using the carotid pressure waveform as a surrogate for aortic pressure is well-established and shown in the previous studies.^46,47^ The FHS data used in this study were composed primarily of White participants of Western European descent with a mean (range) age of 66 (40–91) years. Future studies can aim to include training data from multi-centre data sets to further examine and expand the usage of the proposed spectral regression learning method. The comparison between the parameters derived from the reference wave separation and our proposed pressure-only method is based on cross-sectional data. Future studies could benefit from clinical validation using mortality data and assessing the predictive performance of the pressure-only approach on cardiovascular events.

## Conclusions

This study presented a comprehensive evaluation of a novel spectral regression learning method for pressure-only wave separation analysis in a population-based FHS. We demonstrated the method’s accuracy by comparing wave separation parameters with a reference method employing Doppler ultrasound-derived flow waves and tonometry-measured pressure waveforms. The proposed pressure-only method showed interchangeability with the reference method in a large, heterogeneous cohort and its associations with carotid-femoral PWV as a marker of vascular ageing. Our investigation into carotid-femoral PWV highlighted the significance of forward pressure wave amplitude, with the proposed spectral regression learning method demonstrating similar performance to that of the reference approach. These findings emphasize the clinical applicability and accuracy of the proposed pressure-only wave separation analysis, providing a valuable non-invasive tool for assessing cardiovascular health.

## Supplementary Material

oeae040_Supplementary_Data

## Data Availability

No new clinical data were generated in support of this research. The secondary data analysis codes will be available upon reasonable request to the corresponding author.

## Lead author biography

Arian Aghilinejad, PhD, currently serves as a postdoctoral research associate at the Division of Engineering and Applied Science at the California Institute of Technology (Caltech). He is interested in studying fluid physics in the cardiovascular system and developing engineering-based monitoring solutions for cardiovascular disease patients. He is the recipient of the American Heart Association Fellowship Award, as well as a finalist for the 2024 Young Investigator Award at the American College of Cardiology. He completed his PhD at the University of Southern California and earned his bachelor’s degree from Sharif University of Technology.
