# Supplementary material for: Assessing pressure wave components for aortic stiffness monitoring through spectral regression learning
Source: Eur Heart J Open. 2024 May 21;4(3):oeae040. doi: 10.1093/ehjopen/oeae040 (PMC11165314; doi:10.1093/ehjopen/oeae040)
Supplement: oeae040_Supplementary_Data [file oeae040_supplementary_data.pdf]

Assessing Pressure Wave Components for Aortic Stiffness Monitoring through Spectral Regression Learning

Arian Aghilinejad, PhD[1], Morteza Gharib, PhD[1]

[1]Division of Engineering and Applied Science, California Institute of Technology, Pasadena, CA, United States

Corresponding Author:

Arian Aghilinejad, PhD

1200 E California Blvd, Pasadena, CA, 91125

Email: aghili@caltech.edu

Phone: 302 252 8325

The supplementary material has been provided by the authors to give the readers additional information about their work. This file include:

- **Supplemental Methods**
- **Figures S1 and S2**
- **Tables S1, S2, and S3**

Supplemental Methods

Spectral Regression Learning Method

The first step to implement the spectral regression learning method is to transform data from a high-dimensional input signal space to a low-dimensional space. To do so, we employed Fourier-based decomposition. The Fourier series is a method of combining harmonically related sinusoids and cosines to synthesize a periodic function. Any periodic pressure function *P*(t) can be expressed as a Fourier series with *N* oscillatory components. A typical representation of the Fourier series decomposition is as follows:

$$P(t) = \frac{a_0}{2} + \sum_{n=1}^{N} \left(a_n cos\ \left(\frac{2\pi}{T} nt\right) + b_n sin\ \left(\frac{2\pi}{T} nt\right)\ \right), \tag{1}$$

where *T* is the period of the pressure function *P*(t) (i.e., the cardiac cycle or inverse of HR for blood pressure waveform). In conducting the Fourier decomposition here, we used the sinusoid-cosinusoide form rather than an Amplitude-Phase form to make the inputs independent from each other. This is because the system of all sine and cosine functions at different frequencies builds a complete orthonormal set. Coefficients a_n and b_n are associated with each individual harmonics (cosine and sine) corresponding to different frequencies $f_n = \frac{n}{T}$, and can be calculated by the Fourier transform given by:

$$a_n = \frac{2}{T} \int_0^T P(t) cos\ \left(\frac{2\pi}{T} nt\right) dt, \tag{2}$$

$$b_n = \frac{2}{T} \int_0^T P(t) sin\ \left(\frac{2\pi}{T} nt\right) dt\ , n \epsilon (0,\ \infty). \tag{3}$$

Obtaining the coefficients using Eq. (2) and (3), the represented pressure waveform $\tilde{P}(t)$ with finite selected frequency (i.e., *n = 0 to N*) is achieved by adding up each individual frequency

component (Eq. (1)). In this study, the features of the pressure wave were extracted as the first N^{th} low frequency components of the waveforms using the Fast Fourier Transform (FFT). Previous studies suggested that to fully recover the shape of the pressure waveform using Fourier mode decomposition, the first 20 modes are enough and the error between the measured pressure and the reconstructed after 20 modes is marginal. The selected input features in this study (depending on the chosen number of Fourier modes; *FN*) consists of *FN* cosine coefficients and (*FN - 1)* sine coefficients (sine b_0 is always equal to zero).

In the next step, we use appropriate regression models to build the spectral regression learning model for estimating the Fourier modes of the flow waveform. In this study, the support vector regressor was selected to train and test the regression model due to its proven applicability and performance in previous studies. As a brief summary of this model, a Support Vector Regressor is a machine learning tool utilized for predicting numerical values, such as the amplitude of the sinusoidal-cosinusoidal components of the flow waveform in this study. It operates by identifying a "support vector," essentially a subset of data points crucial for accurate predictions. The Support Vector Regressor aims to create a model with a margin of tolerance on either side of the predicted values by establishing a hyperplane that maximizes the margin between the predicted values and the line. Some points are considered support vectors, indicating their significance in determining the position of this line. SVR is particularly useful when dealing with data that doesn't follow a simple linear pattern, requiring a more flexible approach to capture underlying relationships. This model is trained on features derived from the Fourier decomposition of the pressure waveform, as illustrated in the graphical abstract. Initially, the clinical dataset is divided into training and testing data for all regression learning analyses. The models are exclusively trained on the training population based on the Fourier decomposition of the central pressure waveform and central flow

waveform. The models are trained with the Fourier modes of the pressure waveforms as input and the Fourier modes of the flow waveforms as output. After training, the testing dataset is used only once to assess the model's accuracy. The pressure waveform in the testing dataset is first decomposed into the Fourier modes and fed into the predictive model. The output of the predictive models, representing the estimated modes of the corresponding flow waveform, is then inverse Fourier transformed into the time domain using the computed modes and the length of the signal. The combination of the predictive model and the inverse Fourier transform is referred to as the spectral regression learning model. To implement this model, Python's scikit-learn (sklearn) and TensorFlow packages are employed for training and testing the algorithms. The data split for training and testing is 70% and 30%, respectively. The pandas and numpy packages are also utilized for data processing. The hyperparameters in the models are determined using a ten-fold cross-validation (CV) scheme with the GridSearchCV library.

Forward and Backward Wave Separation Components

In the arterial system, the pressure waveform comprises forward (Pf) and backward (Pb) components. The forward component (Pf) signifies the contribution of forward waves to measured pressure, while the backward component (Pb) represents the contribution of backward waves, resulting in the equation P = Pf + Pb. Pressure wave separation, initially described by Westerhof et al., can be applied to identify the forward and backward components of the measured pressure waveform:

$$Pf = \frac{1}{2}(P + Z_c Q) \tag{4}$$

$$Pb = \frac{1}{2}(P - Z_c Q) \tag{5}$$

Here, Q is the measured flow profile, and Z_c is the characteristic impedance, representing the intrinsic relationship between pressure and flow when waves travel in one direction only. It can be

estimated from measured pressure and flow signals historically achieved in the frequency domain (via Fourier transform). This involves calculating the average ratio of pressure and flow harmonics within a specific frequency range, such as high frequency with the result appearing relatively insensitive to the chosen range (Dujardin and Stone, 1981). Following the computation of forward and backward waves, the maximum amplitude of these waves can define other wave separation parameters. The reflection index (RI) is a significant parameter, defined as:

$$RI = \frac{|Pb|}{|Pb+Pf|} \tag{6}$$

To compute the time delay between the forward and backward waves, half the time of the maximum cross-correlation between Pf and Pb was utilized, following the works of Qasem and Avolio.

Supplemental Figures

Figure S1 demonstrates scatter and Bland-Altman plots indicating the agreement between the measured and estimated uncalibrated averages of the mean flow profile, revealing the method's ability to capture the shape of the flow profile.

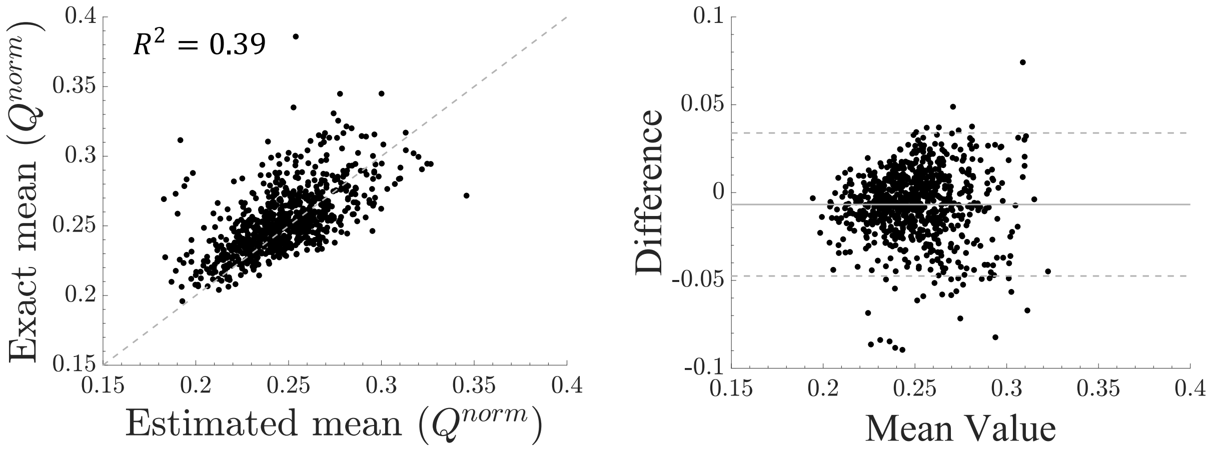

Figure S1 Scatter and Bland-Altman plots indicating the agreement between the measured and estimated uncalibrated averages of the mean flow profile using spectral regression learning method.

Figure S2 demonstrates the distributions of the residuals between reference and estimated variables with respect to age.

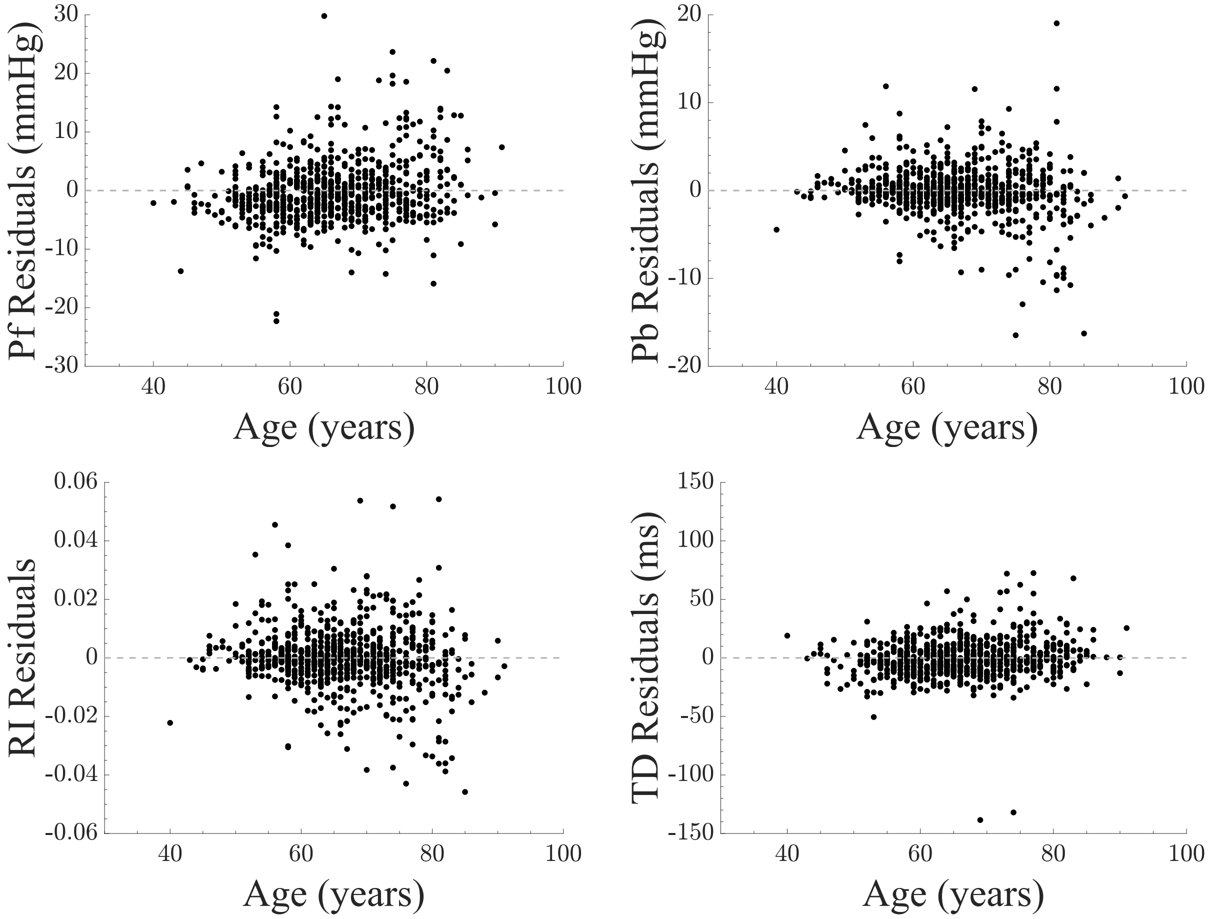

Figure S2 The distributions of the residuals between reference and estimated variables with respect to age for all four variables derived from wave separation analysis including forward wave amplitude (Pf), backward wave amplitude (Pb), reflection index (RI), and time delay (TD) between forward and backward waves.

Supplemental Tables

Table S1 Mean values, errors, and correlations between estimates of uncalibrated (norm) flow parameters using spectral regression learning and reference values (N=753).

Variable	Measured Flow with the Ultrasound	Estimated Flow with SRL
$t_{Q_{max}^{norm}}$(ms)	193 ± 23	191 ± 22
dQ_{max}^{norm}	0.018 ± 0.002	0.0017 ± 0.002
mean(Q^{norm})	0.23 ± 0.03	0.25 ± 0.03
$RMSE[Q_{est}^{norm}$ *vs* $Q_{meas}^{norm}]$ (%)	-	3.00
r [mean(Q_{est}^{norm}) *vs* mean(Q_{meas}^{norm})]	-	0.62
$limit\ of\ Agreement$ [mean(Q_{est}^{norm}) *vs* mean(Q_{meas}^{norm})]	-	0.094
$Mean\ Difference$ [mean(Q_{est}^{norm}) *vs* mean(Q_{meas}^{norm})]	-	0.018

All values are (mean ± SD) except as noted. Q^{norm} denotes the scaled flow waveform from zero to one. SRL indicates spectral regression learning, proposed in this study.

Table S2 Wave separation and hemodynamic parameters per age group.

**Parameters**	Q1 40-52 years	Q2 53-65 years	Q3 66-78 years	Q4 79-91 years
Pf, mmHg	80 ± 10	88 ± 14	95 ± 16	105 ± 20
Pb, mmHg	51 ± 6	55 ± 8	57 ± 8	60 ± 8
TD, ms	78 ± 19	65 ± 18	62 ± 19	57 ± 16
RI	0.39 ± 0.02	0.39 ± 0.02	0.38 ± 0.03	0.36 ± 0.03
PWV	7.6 ± 1.6	9.1 ± 2.5	11.6 ± 3.5	14.5 ± 5.3
Zc, mmHg.s/ml	0.14 ± 0.03	0.17 ± 0.06	0.19 ± 0.07	0.24 ± 0.09
Heart Rate, bpm	61 ± 9	62 ± 10	62 ± 10	62 ± 11

*Q1 includes 44 subjects, Q2 includes 324 subjects, Q3 includes 308 subjects, and Q4 includes 77 subjects.

Table S3 Physiological correlates of the Carotid-femoral pulse wave velocity as a base model (N=753).

**Variables**	β	**SE** (β)	**CI** (β)	P
**Model for Carotid-femoral PWV in the absence of WSA, adjusted $R^2 = 0.383$**				
Sex	1.026	0.324	(0.389, 1.663)	<0.01[+]
Age, years	0.231	0.013	(0.187, 0.239)	<0.001
Mean Blood Pressure, mmHg	0.069	0.009	(0.051, 0.088)	<0.001
Heart Rate, bpm	3.517	0.663	(2.216, 4.818)	<0.001

[+]Indicates a non-significant parameter. PWV and WSA stand for Pulse Wave Velocity and Wave Separation Analysis, respectively.
